# An Effect of Oak-Wood Extract (Robuvit®) on Energy State of Healthy Adults—A Pilot Study

**DOI:** 10.1002/ptr.5368

**Published:** 2015-05-18

**Authors:** Zuzana Országhová, Iveta Waczulíková, Carolina Burki, Peter Rohdewald, Zdeňka Ďuračková

**Affiliations:** 1 Institute of Medical Chemistry, Biochemistry and Clinical Biochemistry, Medical Faculty, Comenius University in BratislavaBratislava, Slovakia; 2 Department of Nuclear Physics and Biophysics, Faculty of Mathematics, Physics and Informatics, Comenius University in BratislavaBratislava, Slovakia; 3 Horphag ResearchAvenue Louis-Casaï 71, 1216, Cointrin/Geneva, Switzerland; 4 Westfälische Wilhelms-Universität Münster, Institut für Pharmazeutische ChemieTwenteweg 15, 48161, Münster, Germany

**Keywords:** Quercus robur, Robuvit, fatigue, ellagitannins, roburins

## Abstract

The purpose of our study was to examine the psychological benefits of the treatment with Robuvit® (Horphag Research Ltd.) – polyphenolic extract obtained from the wood of oak Quercus robur – on the healthy elderly individuals using energy subscale scores of the Activation – Deactivation Adjective Check List. Analysis was focused on the comparison of pre-post treatment effect of Robuvit on symptoms of fatigue. In the total group of volunteers, significant increase of average question scores was found in three of four subscales of feelings (energy, tiredness, and tension) after 4 weeks of Robuvit administration. Effects of extract were observed mainly after stratification of total group of volunteers according to the level of feeling at the pre-treatment questionnaire. Our results demonstrate positive effect of Robuvit on mental and energy level in healthy human without any unwanted side effects. © 2015 The Authors Phytotherapy Research Published by John Wiley & Sons Ltd.

## Introduction

Phenolic phytochemicals exhibit many different positive effects on both, somatic and mental health. (Sasaki *et al.*, 2010; LaRiccia *et al.*, 2008; Mastaloudis and Wood, 2012; Vattem and Shetty, 2005; Ďuračková, 2014).

The French oak wood extract Robuvit® (Horphag Research Ltd.) is a registered proprietary water extract obtained from the wood of *Quercus robur*. The plant belongs to the plant family Fagacae, genus *Quercus*. The oak wood used for Robuvit originates exclusively from oak trees grown in France. Oak wood contains a specific profile of tannins named roburins that are part of the ellagitannins (ETs). Robuvit is standardized and specified to contain at least 20% of roburins (A, B, C, D, E) including grandinin. The two most abundant ETs in the Robuvit are stereoisomers vescalagin and castalagin, which were originally isolated and described by Mayer *et al.* (1967). Roburins and grandinin are dimers of these compounds or differ by the presence of a pentose substituent. They were isolated and identified later by Hervé du Penhoat *et al.* (1991). Further to the roburins and monomeric vescalagin and castalagin, Robuvit contains ellagic acid (EA) as well as gallic acid (Natella *et al.*, 2014).

Owing to their unique molecular structure roburins are very potent antioxidants. Oak wood is currently the only known food source of roburins; thus, the major source of roburins in human diets results from the consumption of wine and spirits (cognac and whiskey) traditionally matured, aged and stored in oak barrels (Glabasnia and Hofmann, 2006).

Bioavailability and biological effects of roburins are still not well known. Robuvit was found out to be bioavailable to humans (Natella *et al.*, 2014), and its consumption is associated with increase of antioxidant capacity at hydrophilic conditions. Influence of Robuvit on antioxidant capacity and antioxidant enzymes was published in our recent paper (Horvathova *et al.*, 2014). Natella *et al.* (2014) identified besides gallic acid and EA also metabolites of ETs named urolithins in the plasma of volunteers after intake of Robuvit. Urolithins are released from ETs by hydrolysis in the intestine by gut microflora (Seeram *et al.*, 2006). In many models, *in vitro* and *in vivo*, the effects of EA as one of compounds of Robuvit have been studying. EA is characterized by antioxidant (Kilic *et al.*, 2014; Kim *et al.*, 2013; Qiu *et al.*, 2013), anticarcinogenic (Chung *et al.*, 2013; Vanella *et al.*, 2013), antiproliferative (Qiu *et al.*, 2013; Loizzo *et al.*, 2009), antiinflammatory (Marín *et al.*, 2013; Rosillo *et al.*, 2012), pro-apoptotic (Larrosa *et al.*, 2006) and antiplatelet properties (Chang *et al.*, 2013). In our recent paper, increased total antioxidant capacity, antioxidant enzymes, superoxide dismutase and glutathione peroxidase, activities as well as decreased level of advance oxidation protein products and lipoperoxides were found after Robuvit administration to adult volunteers (Horvathova *et al.*, 2014).

The aim of our pilot study was to find an effect of natural oak wood extract Robuvit containing a specific profile of tannins named roburins that are part of the ETs with important antioxidant properties on the energy state of elderly healthy volunteers.

## Materials and Methods

### Study subjects

Twenty subjects were recruited from the employees of the Medical Faculty of Comenius University in Bratislava, Slovakia. The study was approved by the Ethical Committee of University Hospital and Faculty of Medicine, Comenius University in Bratislava, Slovakia. All participation was voluntary, and subjects signed informed consent forms prior to participation.

### Inclusion criteria

Eligible subjects were healthy individuals between the ages of 45 and 65 years, free of major medical and mental illnesses, which included AIDS, anaemia, acute inflammatory diseases, renal and cardiovascular disorders, diabetes mellitus, hormone replacement therapy in women, cancer, chronic fatigue syndrome, depression, drug/alcohol dependence, schizophrenia, uncontrolled hypertension as well as requirement of medication prescription. Medical conditions were ascertained by medical interview.

### Fatigue and energy measurement—questionnaires

The fatigue and primary energy state were evaluated by the questionnaire Activation Deactivation Adjective Check List (AD ACL) (Thayer, 1989), a validated instrument that has been used in a wide range of studies including drug and exercise studies (Table1).

**Table 1:** The Activation-Deactivation Adjective Check List (AD ACL) (Thayer, 1989)

Rating scale:				
✔✔	*definitely feel*			
✔	*feel slightly*			
?	*cannot decide*			
no	*definitely do not feel*			
Check list:				
Active	✔✔	✔	?	no
Placid	✔✔	✔	?	no
Sleepy	✔✔	✔	?	no
Jittery	✔✔	✔	?	no
Energetic	✔✔	✔	?	no
Intense	✔✔	✔	?	no
Calm	✔✔	✔	?	no
Tired	✔✔	✔	?	no
Vigorous	✔✔	✔	?	no
At-rest	✔✔	✔	?	no
Drowsy	✔✔	✔	?	no
Fearful	✔✔	✔	?	no
Lively	✔✔	✔	?	no
Still	✔✔	✔	?	no
Wide-awake	✔✔	✔	?	no
Clutched-up	✔✔	✔	?	no
Quiet	✔✔	✔	?	no
Full-of-pep	✔✔	✔	?	no
Tense	✔✔	✔	?	no
Wakeful	✔✔	✔	?	no

Instructions to the user: Each of the words describes feelings or mood. Please use the rating scale next to each word to describe your feelings at this moment.

The AD ACL is a multi-dimensional test of various transitory arousal states, including energetic and tense arousal. AD ACL Short Form consists of 20 self-descriptive adjectives in four subscales (each of five feelings)—energy, tiredness, tension and calmness. The AD ACL is scored by assigning 4, 3, 2, and 1 respectively to the ‘✔✔, ✔, ?’ and ‘no’ scale points, and summing or averaging the five scores for each subscale. In order of appearance, the subscale adjectives are as follows: energetic (active, energetic, vigorous, lively, and full-of-pep), tired (sleepy, tired, drowsy, wide-awake, and wakeful), tension (jittery, intense, fearful, clutched-up, and tense) and calmness (placid, calm, at-rest, still, and quiet). Scoring is based on four possible points for each adjective; thus, the possible score was in the range of 20–80 (each of subscales 5–20). Evaluation of scoring included the reversion of scoring in subscales tiredness and tension; thus, the higher score was considered as better improving effect of Robuvit administration. Volunteers were instructed to rate each adjective in the context of how they felt at the moment they were making their responses. Participants were instructed to fill out the questionnaire at the beginning of study and on each Friday evening for next 6 weeks of study. Our analysis was finally focused on the comparison of pre–post treatment effect of Robuvit on symptoms of fatigue.

### Study design

The study was performed in the course of 8 weeks in three phases. During 2 weeks of ‘run-in period’, volunteers were keeping the diet rules—normal diet with exclusion of antioxidant supplementation as well as important diet source of flavonoids as chocolate, tea, marmalade and others. This diet had to be stuck for a period of the project. During the next 4 weeks (the second—intervention phase), volunteers were administered three times daily by 100 mg of Robuvit (in form of capsule). This period was followed by 2 weeks of wash-out period (the third phase) without Robuvit administration.

Symptoms of fatigue were investigated by AD ACL questionnaire. This questionnaire was filled out by volunteers at home every week (on Friday evening) before the study and six following weeks.

### Statistical analysis of questionnaires

Results for the AD-ACL mood scores (i.e., energy, tiredness, tension and calmness) were based on the weekly self-assessments. We had complete follow-up data on all but one participant. First, we checked for internal consistency within all four dimensions (subscales) using the Cronbach’s alpha coefficient for scale reliability. For scales which are used as research tools to compare groups, alpha values of 0.7 to 0.8 are regarded as satisfactory (Bland and Altman, 1997; Streiner and Norman, 2008; McDowell and Newell, 1996; Cronbach, 1951).

The baseline and follow-up data were summarized using means as measures of central tendency and standard deviations as measures of spread. The distribution pattern of the scores was examined by box-plots for median scores at each time point.

The overall treatment effect was graphically presented as differences in total and subscales between baseline (Q1, questionnaire before intervention phase) and after-treatment (Q5, questionnaire after intervention phase) scores using ladder plots. The degree of association, as well direction and magnitude of the overall effect, was analyzed using simple regression.

Respective time profiles for each subscale were evaluated with a linear regression tool and tested with ANOVA.

The low number of levels for each item, i.e. four response categories according to how the person feels about the questioned item [from definitely no (1) to definitely yes (4)] may not convey enough information cues to support efficient recognition of time profiles of the mean scores. In order to remove variability (which cannot be outweighed by the combination of sample and effect size), we have focused on the comparisons of the baseline scores with either the post-treatment or after-washout-period scores. The differences in counts across response categories were tested using chi-squared statistical tests. Between-group differences in subscale summary scores were tested using the paired *t*-test followed by the agreement analysis.

Analyses were carried out using StatsDirect statistical software, version 2.7.8. A two-tailed alpha level of 0.05 was used to determine statistical significance.

## Results

The purpose of this study was to examine the psychological benefits of the treatment with Robuvit using questionnaire AD ACL.

As the questionnaire AD ACL was not validated in Slovakia yet, we have evaluated its internal consistency. Our results showed that the overall reliability of our instrument—designed as a research tool to compare related data (prior-after treatment) in the participants—was good (except subscale calmness), the alpha values ranged from 0.75 to 0.86 at the baseline, and from 0.77 to 0.84 after treatment.

**Figure 1:**
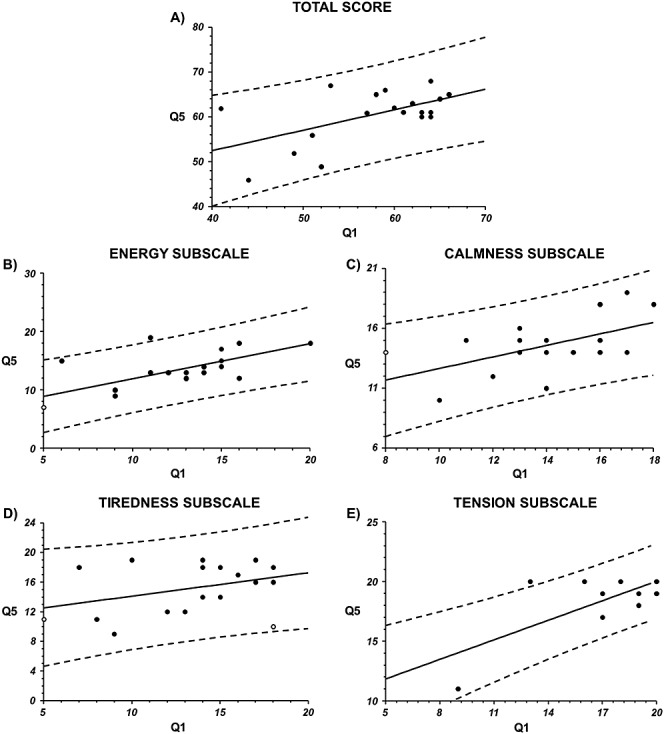
Correlation of paired data for Q1 versus Q5, simple linear regression. (a) For total score in Q1 versus Q5 (points in the range of 20–80); *r* = 0.5704, *p* = 0.0108. (b) Subscale energy (points in the range of 5–20); *r* = 0.6829, *p* = 0.0013. (c) Subscale calmness (points in the range of 5–20); *r* = 0.5498, *p* = 0.0147. (d) Subscale tiredness (points in the range of 5–20); *r* = 0.3703, *p* = 0.1186. (e) Subscale tension (points in the range of 5–20); *r* = 0.7555, *p* = 0.0002. Q1, questionnaire before intervention phase; Q5, questionnaire after intervention phase.

According to our expectations, the volunteers’ responses were correlated. It is visible in correlation of total sums of points in Q1 (questionnaire no. 1—before Robuvit administration) and Q5 (questionnaire no. 5—after 4-week administration of Robuvit) (range of points is 20–80) as well as in correlations of total sums in individual subscales in Q1 and Q5 (range of points is 5–20). (Fig. 1)

### Comparative analysis of average counts across response categories

In comparative analysis of average counts across response categories (subscales) in Q1 (questionnaire no. 1—before Robuvit administration) and Q5 (questionnaire no. 5–after 4 weeks of Robuvit administration), we have found that effects of subscales are significantly differing from each other (*p* = 0.0011). Effect of Robuvit administration respectively (time) is also significant; average scores in Q5 was increased (*p* = 0.0043) compared with Q1.

**Average score for the question** (feeling) of individual subscales of feelings in total sample (all volunteers) was significantly increased in three of four subscales: Subscale energy average score was increased by 8.8%, subscale tiredness by 12% and subscale tension by 4,16% compared with baseline values (Table2). The positive effects of Robuvit on fatigue can be expressed as a sum of changes of significant improvements compared with baseline as 25.11%.

**Table 2:** Comparative analysis of average question scores (1–4 points) reached in questionnaires for individual subscales in total sample (all volunteers) (subscales tiredness and tension after reversion)

Subscale	Mean quest 1	Mean quest 5	Change by points	Change by %	*p*
Energy	2.400	2.611	0.211	8.8	0.0176
Calmness	2.789	2.905	0.116	4.16	NS (0.2292)
Tiredness	2.716	3.042	0.326	12	0.0070
Tension	3.663	3.821	0.158	4.31	0.0183

In comparison of total scores (maximum of 80 points) reached in the questionnaires, we have found marginally significant increase by 4.05 points after 4 weeks of Robuvit administration (*p* = 0.0905). In analysis of total score of subscales (maximum of 20 points), this trend was confirmed only in subscale tension with increase of score 0.79 point (*p* = 0.0917).

It was presumable that the participants who scored their feeling in the items at the top of scale have not shown an effect size with the same chance as those who scored their feeling at lower level. Therefore, we stratified the participants into two groups, those with a summary subscale score below 14 points and those who achieved 14 and more points at the baseline from possible 20 points. For the total score, a 60-point result was chosen as a cut-off-point (20–59 the first subgroup and 60–80 was the second one).

**Figure 2:**
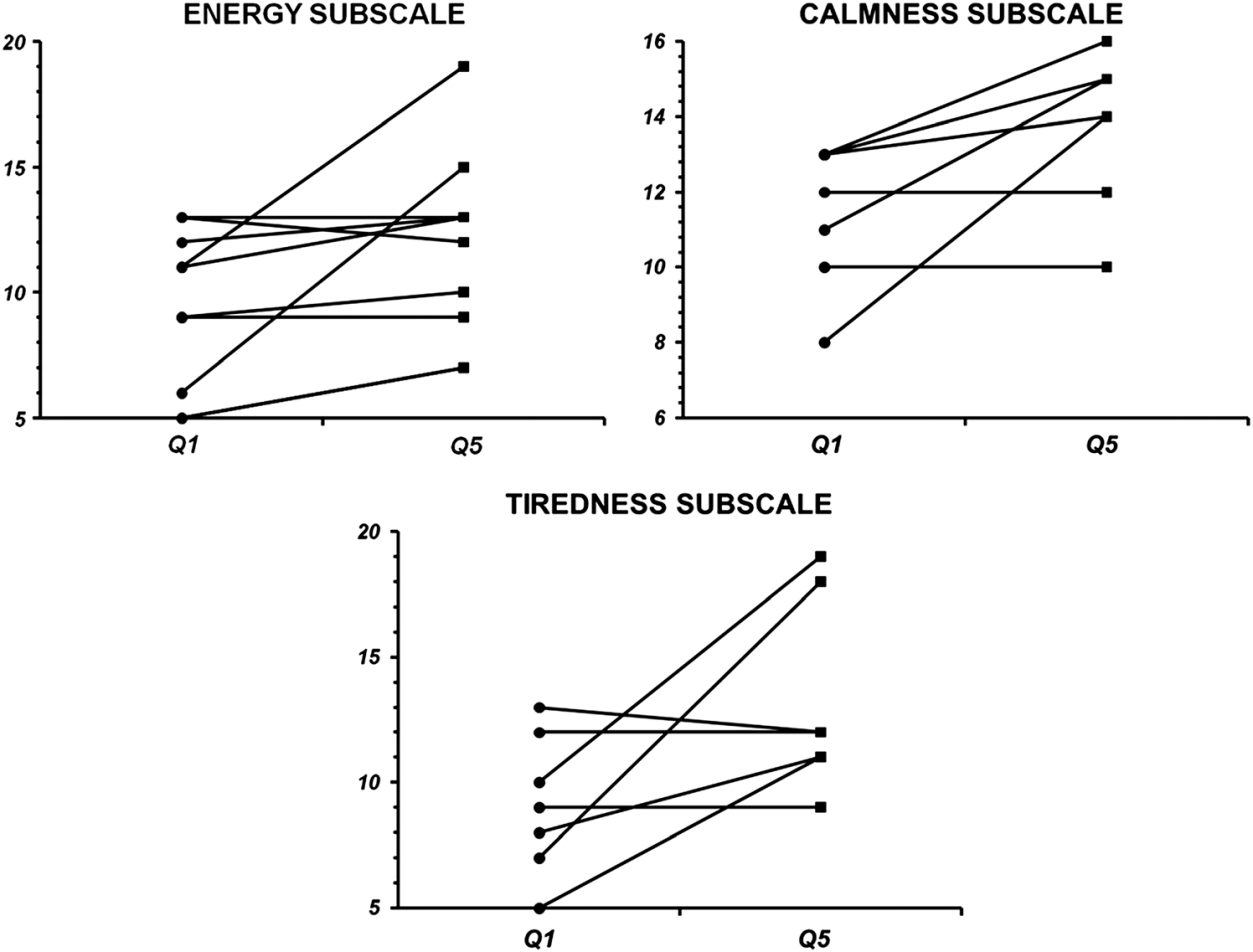
Comparison of total scores reached in the individual questionnaire subscales (5–20 points) reached in subgroup with baseline < 14 points (subscales tiredness and tension after reversion). Q1, questionnaire before intervention phase; Q5, questionnaire after intervention phase.

Using stratification, we have found that in volunteers who have started in Q1 with lower total score, there was visible improvement by 8.1 points in average (one sided *p* = 0.0491—as we have predicted effect of administration of Robuvit as forward hypothesis) after 4 weeks of Robuvit administration. In subscales, stratification has shown improving effect of Robuvit administration again only in the groups with lower baseline—significant in the subscale calmness and marginally significant in the subscales energy and tiredness (Fig. 2 and Table3)

**Table 3:** Comparative analysis of total scores reached in questionnaires using stratification of data in the group with baseline < 14 points (total scores in individual subscales—range 5–20 points) (subscales tiredness and tension after reversion)

Subscale	Change (points)	*p*
Energy	↑ by 2.18 (*n* = 11)	0.051
Calmness	↑ by 2.29 (*n* = 11)	0.0342
Tiredness	↑ by 4.0 (*n* = 7)	0.0679
Tension	− (*n* = 2)	—

### Safety

No unwanted side effects were observed neither during 4-week consumption of the oak wood extract by the volunteers nor after 2 weeks of wash-out period.

## Discussion

The purpose of this study was to examine the psychological benefits of the treatment with Robuvit on the healthy elderly individuals. The analysis was finally focused on the comparison of pre–post treatment effect of Robuvit on symptoms of fatigue using the AD ACL as the internal consistency of this questionnaire was confirmed.

In the total group of volunteers, significant increase of average question scores was found in three of four subscales of feelings (energy, tiredness and tension) after 4 weeks of Robuvit administration. Effects of extract were observed mainly after stratification of total group of volunteers according to the level of feeling in the items at the pre-treatment questionnaire. Using stratification, we have found visible improvement in total subscale score in volunteers who have started with lower (worse) total score. Improving effect of Robuvit was marginally significant in energy and tiredness subscales in volunteers with lower baseline of feelings scoring.

A large number of phytochemical containing nutraceuticals with various compositions including ellagitanins are investigated. However, the scientific evidence supporting their health benefits is still insufficient, and it is mostly based on in vitro or animal model assays (Espín *et al.*, 2007).

The natural oak wood extract Robuvit containing a specific profile of tannins named roburins that are part of the ETs has shown important *in vitro* antioxidant properties. Belcaro *et al.* (2014) found that extract from oak wood could reduce plasma free radicals in peripheral blood in patients suffering from chronic fatigue syndrome. Because of observations that reduction of oxidative stress was associated with an improvement of cognitive function, the influence of Robuvit on symptoms of chronic fatigue syndrome was also evaluated in their study. The reduction of oxidative stress markers advance oxidation protein products and lipoperoxides in serum as well as increasing of total antioxidant capacity and activities of antioxidant enzymes, Cu/Zn superoxide dismutase and catalase in red blood cells was observed and published in our recent paper (Horvathova *et al.*, 2014).

Contrary to Belcaro *et al.* (2014), who has found improvement of mood in chronic fatigue syndrome patients administered with Robuvit 200 mg daily for 6 months, we have found significant mood improvement in volunteers without fatigue syndrome—after 4-week administration of Robuvit in higher dose (300 mg/day). Similar effect to our and Belcaro studies was observed in two clinical studies with another antioxidant food supplement, maritime pine bark extract Pycnogenol, where psychic well-being in menopausal women was also increased (Errichi *et al.*, 2011; Yang *et al.*, 2007). Our results demonstrate positive effect of Robuvit on mental and energy state and its possible efficiency as a dietary supplement in healthy human without any unwanted side-effects.
